# Genetically Engineered Hematopoietic Stem Cells Deliver TGF‐*β* Inhibitor to Enhance Bone Metastases Immunotherapy

**DOI:** 10.1002/advs.202201451

**Published:** 2022-08-10

**Authors:** Beilei Wang, Jinyu Bai, Bo Tian, Hao Chen, Qianyu Yang, Yitong Chen, Jialu Xu, Yue Zhang, Huaxing Dai, Qingle Ma, Ziying Fei, Heng Wang, Fang Xu, Xiaozhong Zhou, Chao Wang

**Affiliations:** ^1^ Institute of Functional Nano & Soft Materials (FUNSOM), Jiangsu Key Laboratory for Carbon‐Based Functional Materials & Devices Soochow University 199 Ren'ai Road Suzhou Jiangsu 215123 China; ^2^ Department of Orthopedics The Second Affiliated Hospital of Soochow University Suzhou Jiangsu 215004 China

**Keywords:** bone metastases, genetically engineering, hematopoietic stem cell, immune checkpoint blockade therapy, transforming growth factor beta inhibitor

## Abstract

Owing to the immune microenvironment of bones and low selectivity of the drug, patients with bone metastases often respond poorly to immunotherapy. In this study, programmed cell death protein 1 (PD1)‐expressing hematopoietic stem cells (HSCs) are genetically engineered for bone‐targeted delivery of the transforming growth factor beta (TGF‐*β*) small‐molecule inhibitor SB‐505124 (SB@HSCs‐PD‐1). Intriguingly, compared to anti‐PD‐L1 monoclonal antibodies, as “living drugs”, HSCs‐PD‐1 not only show great targeting ability to the bone marrow, but are also able to reduplicate themselves within the bone marrow niche and continuously express PD‐1 molecules. The SB released from HSCs‐PD‐1 competitively bound to TGF‐*β* receptors on CD4^+^ T cells and facilitate CD4^+^ T cell differentiation to helper T (T_H_)1 and T_H_2 cells, thereby reprogramming the local immunosuppressive milieu of the bone marrow. Additionally, HSCs‐PD‐1 can block programmed death‐ligand 1 on tumor and myeloid cells, resulting in reinvigorated anti‐tumor immunity of T cells. In conclusion, in the present study, an alternative cell engineering strategy is delineated for immune checkpoint blockade therapy, to target bone metastasis using HSCs as a platform, which shows great promise in the treatment of bone metastases.

## Graphical abstract

High‐expressing programmed death‐1 hematopoietic stem cells (HSCs‐PD‐1) are prepared for bone‐targeted delivery of the transforming growth factor beta small‐molecule inhibitor SB‐505124, called SB@HSCs‐PD‐1. It shows great bone‐targeting ability and self‐replitcate capacity in the bone marrow, therapy reprogramming the local immunosuppressive milieu of the bone metastases, which provides a universal platform technique for the bone disease.

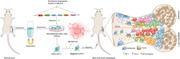

## 1 Introduction

Bone is one of the most common metastatic destinations in breast, prostate, and lung cancer.^[^ ^1^ ^]^ In the case of metastatic breast cancer, up to 45% of patients have their first metastasis in the bones.^[^ ^2^ ^]^ Approximately 70%–80% of metastatic castration‐resistant prostate cancer (CRPC) metastasizes to the bones, which far exceeds the frequency of metastasis to other organs.^[^ ^3^ ^]^ More importantly, more than two‐thirds of bone metastases are not confined to the bone, but further metastasize to systemic metastases, ultimately leading to patient mortality.^[^ ^4^ ^]^ Bone metastases are often accompanied by bone destruction and drastic bone pain, thereby severely impairing the quality of life of the patients.^[^ ^5^ ^]^

Immune checkpoint blockade (ICB) therapy is a cancer immunotherapy that reactivates the immune system, by blocking immune checkpoint molecules, and thereby allowing for an attack on tumor cells.^[^ ^6^ ^]^ The past decade has witnessed a revolution in the treatment of metastasis using ICB therapy. Despite the encouraging clinical results, many patients with bone metastases are often ineffective and refractory to ICB, which could be attributed to a great extent to the obstacles imposed by the immune microenvironment of the bone.^[^ ^7^ ^]^ The large amounts of immature and inhibitory immune cells in the bone marrow make the bone a particularly immune‐privileged area, with suppressed cytotoxic T lymphocytes, compared to other organs.^[^ ^8^ ^]^ In a phase III trial, the responsiveness of patients with CRPC bone metastases to ICB was much poorer than that of patients without CRPC bone metastases.^[^ ^9^ ^]^ Compared with subcutaneous tumors, the tumor immune microenvironment of bone metastases is characterized by dramatically elevated transforming growth factor‐*β* (TGF‐*β*), which drives CD4^+^ T cells to polarize to the T‐helper (T_H_)17 lineage, rather than the T_H_1 lineage.^[^ ^7^, ^10^ ^]^ TGF‐*β* in bone metastases is secreted not only by tumor cells, but also by the destroyed bone matrix, which is a major reservoir of TGF‐*β*.^[^ ^11^ ^]^ High levels of TGF‐*β* compromise the ability of bone metastases to respond to ICB therapy.

In advanced stages of cancer, TGF‐*β* secreted by tumor cells and tumor‐associated macrophages induce tumor invasion, metastasis, and fibrosis.^[^ ^12^ ^]^ More seriously, TGF‐*β* is a crucial physiological immunosuppressor in humans.^[^ ^13^ ^]^ The immunosuppressive response to TGF‐*β* allows tumors to evade the anticancer immune response. Hence, TGF‐*β* has attracted considerable attention as a new target for tumor immunotherapy, and the development of related blockers is constantly emerging.^[^ ^7^, ^14^ ^]^ Several small‐molecule inhibitors of TGF‐*β*, such as halofuginone and galunisertib, are effective in limiting the progression of bone metastases, but the potential toxicity of these small‐molecule inhibitors has not yet been fully evaluated.^[^ ^15^, ^16^ ^]^ In addition, GlaxoSmithKline and Merck jointly developed the programmed death‐ligand 1 (PD‐L1)/TGF‐*β* double‐antibody, Bintrafusp alfa (M7824), which suppresses metastasis in multiple preclinical models^[^ ^17^ ^]^ and clinical trials^[^ ^18^ ^]^ than either a TGF‐*β* trap or an anti‐PD‐L1 antibody alone. However, in a phase 1 trial to treat patients with non‐small cell lung cancer, the overall response rate (21.3%), and irAEs rate (69%) required continuous improvement.^[^ ^19^ ^]^ Therefore, further efforts are needed to develop targeted delivery systems that increase the overall response rates and reduce the irAEs rate.

Cell‐based drug delivery platforms have recently emerged as natural systems, owing to their inherent biological compatibility.^[^ ^14^, ^20^ ^]^ Compared to nanoparticles and nanovesicles,^[^ ^21^ ^]^ living cell‐based delivery platforms can self‐proliferate and differentiate at the targeted site, making them living drugs that work for long term. Given the effect of homing on the bone marrow as well as their low immunogenicity, hematopoietic stem cells (HSCs) show great potential for bone metastases treatment. In this study, we prepared genetically engineered HSCs with high programmed death‐1 (PD‐1) expression, for bone‐targeted delivery of the TGF‐*β* small‐molecule inhibitor SB‐505124 (SB), which will hereafter be referred to as SB@HSCs‐PD‐1. The bone‐targeting ability of SB@HSCs‐PD‐1 was observed in mice with bone metastases. On the one hand, SB released from HSCs‐PD‐1 competitively bound to TGF‐*β* receptors on CD4^+^ T cells and facilitated differentiation of CD4^+^ T cells into T helper (T_H_)1 and T_H_2 cells, thereby reprogramming the local immune milieu of the bone marrow. In contrast, HSCs‐PD‐1 blocked PD‐L1 on tumor and myeloid cells, resulting in reinvigorated anti‐tumor immunity of the T cells. More importantly, compared to anti‐PD‐L1 monoclonal antibodies (mAbs), as “living drugs”, HSCs‐PD‐1 were able to reduplicate themselves within the bone marrow niche (but not other accumulation organs) for at least 8 d post‐injection, and continuously express PD‐1 molecules. Successful reversal of the immunosuppressive microenvironment in metastatic bone tumors was achieved using the SB@HSCs‐PD‐1 platform. In addition, our results demonstrated a better therapeutic outcome than the PD‐L1 antibody, for the treatment of bone metastases in animal models.

## 2 Results

### 2.1 Identification of the Immune Microenvironment of Bone Metastasis

**Figure 1:**
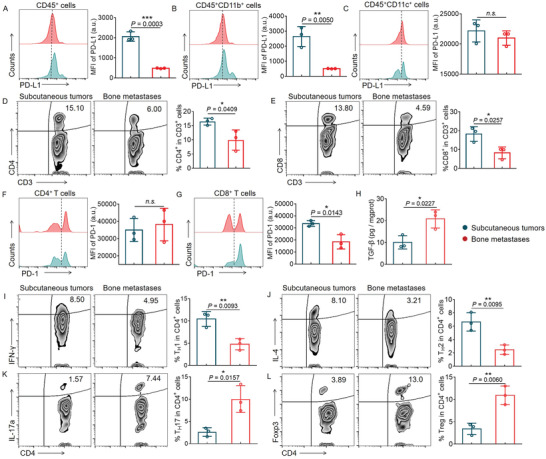
Comparing the immune microenvironment of subcutaneous tumors and bone metastases. A–C) Comparison of the expression level of PD‐L1 in CD45^+^ (A), CD45^+^CD11b^+^ (B), and CD45^+^CD11c^+^ (C) cells in subcutaneous tumors and bone metastases. D,E) The proportions of CD4^+^ (D) and CD8^+^ (E) T cells in subcutaneous tumors and bone metastases, respectively. F,G) Comparison of the expression level of PD‐1 in CD4^+^ (F) and CD8^+^ (G) T cells in subcutaneous tumors and bone metastases. H) Quantification of TGF‐*β* levels in subcutaneous tumors and bone metastases. I–L) The proportion of CD4 T_H_ lineages, T_H_1 (I), T_H_2 (J), T_H_17 (K), and T_reg_ (L), in bone metastases and subcutaneous tumors. Data have been represented as mean ± SD. Statistical significance was calculated using a two‐tailed unpaired Student's *t*‐test (n = 3), *P*‐value: *P<0.05, **P<0.01, and ***P<0.001; n.s., no significance; a.u., arbitrary units; MFI, mean fluorescence intensity.

To confirm that bone metastasis is refractory to ICB, we induced bone metastases in C57BL/6 mice, via femoral inoculation of luciferase‐labeled B16F10 melanoma cells (B16F10‐Luc)^[^ ^5a^ ^]^ and prepared a subcutaneous tumor model as a control. Ten days later, bone metastases in the left femur and distant lung metastases were observed after the dissection of the mice, indicating their high aggressiveness in the bone marrow (Figure S1A, Supporting Information). Compared to subcutaneous tumors, the expression levels of PD‐L1 were reduced in both CD45^+^ and CD45^+^CD11b^+^ cells of bone metastases (**Figure** **1**A,B), while there was no obvious change in CD45^+^CD11c^+^ cells (Figure 1C). The low level of PD‐L1 in myeloid cells within bone metastases is another potential reason for their refractory response to ICB, indicating that a combination therapy strategy should be applied for the treatment of bone metastases. In parallel, we found that the ratios and numbers of CD4^+^ T cells and CD8^+^ T cells were reduced within bone metastases, compared to those in subcutaneous tumors (Figure 1D,E). Although the PD‐1 level of CD4^+^ T cells in bone metastases was similar to that in subcutaneous tumors (Figure 1F), it was expressed at significantly lower levels in CD8^+^ T cells in bone metastases (Figure 1G). While Tim‐3 and Lag‐3 expressions were not changed significantly between the two tumor types (Figure S1B,C, Supporting Information). These results suggested that CD8^+^ T cells were hyporesponsive to ICB therapy in bone metastases compared to subcutaneous tumors, due to the lacking expression of PD‐1.^[^ ^22^ ^]^ Studies have shown that excessive secretion of TGF‐*β* in bone metastases hinders adaptive immunity, leading to resistance to immunotherapy.^[^ ^7^ ^]^ We observed a significantly elevated TGF‐*β* level in bone metastases, compared to that in subcutaneous tumors (Figure 1H). In addition, lower proportions of T_H_1 effector cells [interferon gamma (IFN‐*γ*)^+^] and T_H_2 cells [(interleukin (IL)‐4^+^], as well as enhanced frequencies of T_H_17 cells (IL‐17a^+^) and T_reg_ cells (Forkhead box protein 3, Foxp3^+^), were detected in bone metastases (Figure 1I,L). Our results indicated that elevated TGF‐*β* levels in the bone marrow play a pivotal role in creating an immunosuppressive microenvironment that facilitates disseminated tumor cell growth.^[^ ^6c^ ^]^

### 2.2 Preparation of HSCs Loaded with TGF‐*β* Inhibitors

**Figure 2:**
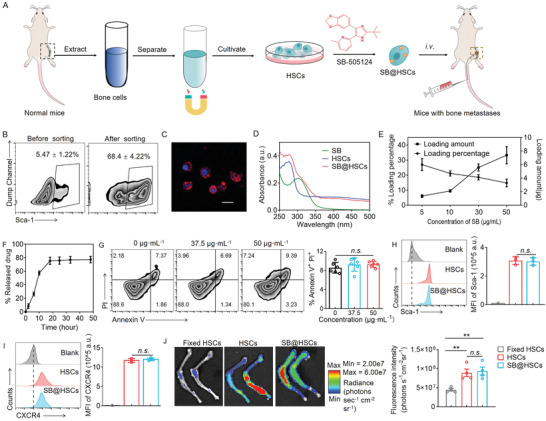
Characterization of HSCs loaded with TGF‐*β* inhibitors. A) Scheme of the preparation process and treatment method for SB@HSCs. B) Purity of the HSCs was assessed using flow cytometry. C) Morphology of the HSCs was observed using confocal microscopy. Scale bar: 10 µm. D) Representative UV–vis absorption peaks of SB, HSCs, and SB@HSCs in PBS. E,F) The loading amount and percentage (E) as well as in vitro cumulative release profile (F) of SB from SB@HSCs (n = 3). G) The effect of SB at different concentrations on HSC apoptosis (n = 6). H,I) Expression levels of Sca‐1 (H) and CXCR4 (I) in HSCs and SB@HSCs were analyzed using flow cytometry (n = 3). J) Representative fluorescence images and quantitative analysis of bone isolated 4 h after injection of DiD‐labeled paraformaldehyde‐fixed HSCs, HSCs, and SB@HSCs (n = 4). Data have been represented as mean ± SD. Statistical significance was calculated using Student's *t*‐test and one‐way ANOVA followed by Tukey's post‐hoc test; *P*‐value: **P<0.01; n.s., no significance; a.u., arbitrary units; MFI, mean fluorescence intensity.

Blocking TGF‐*β* signaling in CD4^+^ T cells can alleviate immunosuppression and improve regression of bone metastases.^[^ ^14^, ^23^ ^]^ However, the development of TGF‐*β* inhibitors (small‐molecules or antibodies) has been accompanied by the issues of low selectivity and toxicity.^[^ ^17^, ^24^ ^]^ Therefore, we designed a bone‐targeting platform using bone‐derived HSCs, to deliver TGF‐*β* inhibitors (**Figure** **2**A). Stem cell antigen‐1 (Sca‐1) is a stem cell antigen marker, and C‐X‐C chemokine receptor (CXCR)4 is the key factor that mediates bone homing and engraftment.^[^ ^25^ ^]^ HSCs were obtained from mice and purified using magnetic beads coupled with an anti‐Sca‐1 antibody, to achieve a purity of 68.4% ± 4.22% (Figure 2B,C). We further found that the expression level of CXCR4 in sorted Sca‐1^−^ cells were significantly higher than that in unsorted cells (Figure S2A, Supporting Information). The small‐molecule TGF‐*β* inhibitor SB‐505124 could be easily loaded onto HSCs, due to the hydrophobic interaction between SB and the cytomembrane of HSCs. The absorption spectrum of SB@HSCs indicated the successful loading of SB into the HSCs (Figure 2D). The loading percentage of SB was approximately 14.78% ± 2.55%, and 7.39 ± 0.49 µg SB could be loaded into 1×10^5^ cells (Figure 2E). SB was released from the SB@HSCs in a sustained manner, and 77.24% ± 4.75% of SB was released within 48 h in phosphate‐buffered saline (PBS), at 37 °C, as determined using high‐performance liquid chromatography (HPLC) (Figure 2F). The loading of SB at different concentrations showed little cytotoxicity towards HSCs (Figure 2G and Figure S2B, Supporting Information). In addition, we explored the effect of SB on HSCs. Sca‐1, c‐kit, and CD44 have been adopted as markers of HSCs while CXCR‐4 in HSCs is considered as a key homing factor. We found no significant difference in the expression of Sca‐1, CXCR4, c‐kit, and CD44 in SB@HSCs, compared to that in naive HSCs (Figure 2H,I and Figure S2C,D, Supporting Information). Next, we assessed the homing of these HSCs, compared to that of paraformaldehyde‐fixed dead HSCs. An in vivo imaging system (IVIS) demonstrated that HSCs exhibited greater accumulation efficiency in the bone marrow than in the paraformaldehyde‐fixed HSCs, suggesting that HSCs have a great capacity to home towards bone‐marrow niches (Figure 2J). SB loading did not significantly affect the homing ability of the HSCs (Figure 2J). We also explored the bone targeting of SB@HSCs within 24 h, and the fluorescence intensity of DiD‐labeled SB@HSCs in bones peaked around 1–4 h (Figure S3A, Supporting Information). The rapid homing of intravenously injected SB@HSCs to the bone marrow within hours reduces the release of SB into the circulation. The subsequent decrease in fluorescence intensity may be related to the loss of DiD dye (Figure S3A, Supporting Information). We further analyzed the homing efficiency of SB@HSCs in the bone marrow. The highest efficiency was around 1 h (≈3.81%), and remained stable basically as determined by the ex vivo fluorescence image (Figure S3B, Supporting Information). The homing efficiency of SB@HSCs to the bone marrow at 24 h post‐administration was 7.94‐fold that of the free dye DiD group (Figure S3C, Supporting Information).

### 2.3 SB@HSCs Reverse the Immunosuppressive Microenvironment in Bone Metastases

**Figure 3:**
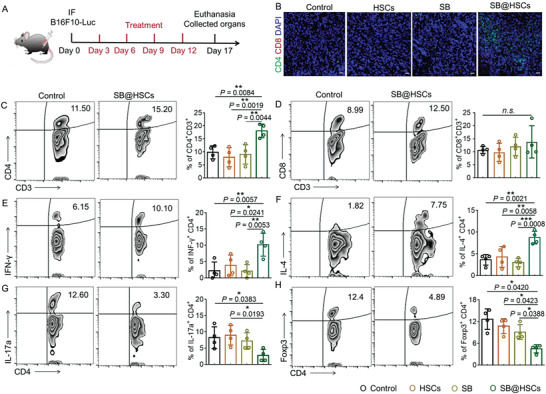
SB@HSCs reverse the immunosuppressive microenvironment in bone metastases. A) Schematic illustration of therapy. Mice were intra‐femorally injected with 1×10^6^ B16F10‐Luc cells and then treated with PBS, HSCs (1×10^5^ cells/mouse), SB (7.5 µg/mouse), SB@HSCs (1×10^5^ cells/mouse). B) Representative immunofluorescence images of CD4 (green) and CD8 (red) in bone metastases, after different treatments. Scale bar: 20 µm. C–H) Representative flow cytometry zebra plots and statistical analyses of immune cells in mice, after different treatments. CD4^+^ T (C), CD8^+^ T (D), T_H_1 (E), T_H_2 (F), T_H_17 (G), and T_reg_ (H) cells (n = 4). Data have been represented as mean ± SD. Statistical significance was calculated using one‐way ANOVA followed by Tukey's post‐hoc test; *P*‐value: *P<0.05, **P<0.01, and ***P<0.001; n.s., no significance; IF, intra‐femoral injection; a.u., arbitrary units; MFI, mean fluorescence intensity.

To test whether SB@HSCs reshape the immune landscape in the bone metastasis microenvironment, we treated bone metastatic mice with SB@HSCs, as shown in **Figure** **3**A. Upon intravenous administration of SB@HSCs into the bone metastatic mice, there was an appreciable increase in the infiltration of CD45^+^ cells into the tumor tissue, as compared to that upon free SB administration (Figure S4A, Supporting Information). However, macrophage polarization was not significantly affected by the SB@HSCs treatment (Figure S4B, Supporting Information). Although the PD‐1 level of CD4^+^ T cells did not change significantly (Figure S4C, Supporting Information), it was expressed at higher levels in CD8^+^ T cells after SB@HSCs treatment (Figure S4D, Supporting Information). In addition, CD4^+^ T cells infiltrated more into the bone marrow of mice receiving the SB@HSCs, while there was no significant change in the number of CD8^+^ T cells (Figure 3B–D and Figure S5A,B, Supporting Information). Next, we investigated the population of CD4^+^ T cells within the tumor immune microenvironment of bone metastasis. As expected, compared to free SB administration, SB@HSC treatment remarkably increased the frequency of T_H_1 and T_H_2 cells, while notably lowering the ratio of T_H_17 to T_reg_ cells (Figure 3E–H and Figure S5C–F, Supporting Information). Together, these results suggested that the targeted delivery of SB by means of HSCs effectively reshapes the bone metastatic environment, by forcing CD4^+^ T cells into effector CD4^+^ T cells, rather than regulatory CD4^+^ T cells. Notably, there was a significant increase in the PD‐L1 expression in tumor cells, after treatment with SB@HSCs (Figure S5G, Supporting Information).

### 2.4 Therapeutic Response to Anti‐PD‐L1 Blockade with SB@HSCs

**Figure 4:**
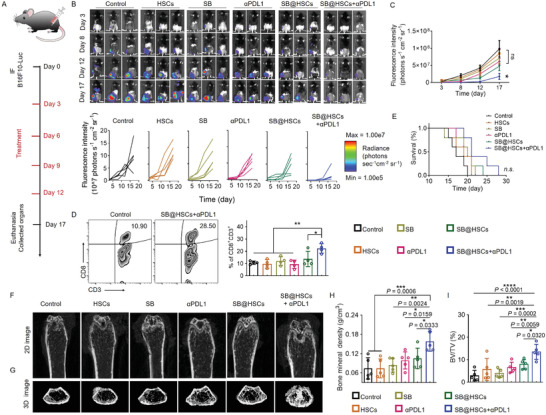
The therapeutic response to anti‐PD‐L1 with SB@HSCs. A) Schematic illustration of therapy. Mice were intra‐femorally injected with 1×10^6^ B16F10‐Luc cells and then treated with PBS (n = 5), HSCs (n = 5), SB (n = 5), anti‐PD‐L1 (n = 5), SB@HSCs (n = 5), or SB@HSCs combined with anti‐PD‐L1 (SB@HSCs+*α*PD‐L1) (n = 5). B) Representative IVIS images and individual tumor growth kinetics of B16F10‐Luc tumors in each group. C) Average tumor growth of mice after different treatments. D) Representative flow cytometry zebra plots of cells and statistical analyses of CD8^+^ T cells in mice, after different treatments (n = 4). E) Survival curve of mice with different treatments. F,G) Representative micro‐CT 2D images (F) and 3D reconstruction images (G) of the distal part of tumor‐bearing femurs in the different groups. H,I) Quantification of BMD (H) and BV/TV (I) after surgery (n = 5). Data in Figure 4C have been represented as mean ± SEM, while data in the other figures have been represented as mean ± SD. Statistical significance was calculated using one‐way ANOVA followed by Tukey's post‐hoc test; *P*‐value: *P<0.05, **P<0.01, ***P<0.001, and ****P<0.0001. The data in Figure 4E were analyzed using the log‐rank (Mantel–Cox) test; n.s., no significance; IF, intra‐femoral injection.

These results prompted us to test whether SB@HSCs could synergize with PD‐L1 blockade to induce anti‐tumor immunity and regression of bone metastasis. The treatment process is shown in **Figure** **4**A. Bone metastatic mice were treated with PBS, HSCs, SB, anti‐PD‐L1 monoclonal antibodies (mAbs), SB@HSCs, or combination therapy once every three days, for a total of four administrations. Bone metastasis growth was assessed over time using IVIS and quantitative analyses (Figure 4B,C). As expected, the bioluminescence signal of bone metastases increased rapidly in mice treated with PBS, HSCs, or SB alone. Consistent with a previous study, we observed the complete abrogation of the therapeutic response to anti‐PD‐L1 in bone metastasis‐bearing mice. Mice treated with SB@HSCs had a mildly lower metastatic burden in their bone than control mice, while combination therapy significantly contributed to bone metastases regression (Figure 4C). Treatment with SB@HSCs in combination with anti‐PD‐L1 blockade resulted in further enhanced intratumoral CD8^+^ T cell responses, as compared to that seen in the controls (Figure 4D). However, as the B16F10 bone metastatic tumor was quite aggressive, we did not observe a significant survival benefit of our combination therapy strategy in mice with bone metastases; on the other hand, there was an improvement in the median survival of mice receiving SB@HSCs and anti‐PD‐L1 mAbs, as compared to that of the controls (Figure 4E).

Previous studies have reported that bone metastases result in osteolytic bone lesions, by activating osteoclasts, which are accompanied by the advanced secretion of TGF‐*β* from osteoclasts.^[^ ^5^, ^26^ ^]^ Therefore, we randomly selected bone samples from each group, to assess bone destruction using micro‐computed tomography (micro‐CT). 2D images and 3D reconstruction showed that as compared to that in the control groups, the trabecular bone structure of mice in the combined treatment group was less damaged by the tumor (Figure 4F,G). In addition, the bone mineral density (BMD) and bone tissue volume per total tissue volume (BV/TV) in these mice were significantly higher than those in the control groups, suggesting that combination therapy markedly inhibited bone destruction (Figure 4H,I). Collectively, our strategy of using HSCs as a platform for SB bone‐targeting delivery resulted in an improved response of bone metastasis to anti‐PD‐L1 therapy.

### 2.5 Generation of Genetically Engineered HSCs to Deliver SB

**Figure 5:**
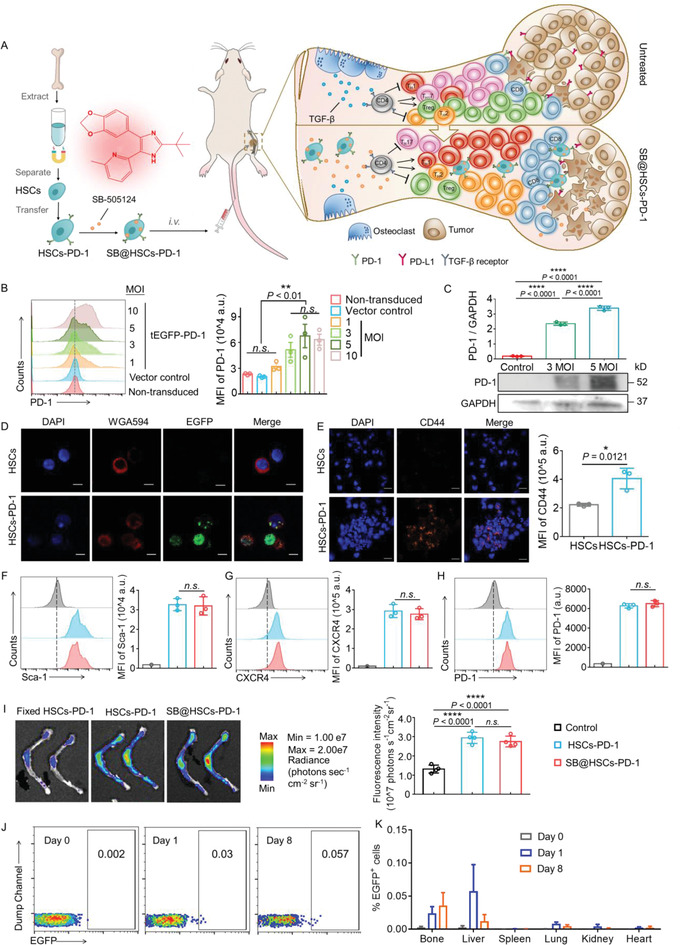
Generation of genetically engineered HSCs to deliver SB. A) Schematic preparation and mechanism of the SB@HSCs‐PD‐1 therapy for bone metastases. B) Flow cytometric analysis of the lentivirus transfection efficiency at various MOIs, after 48 h of co‐culture with HSCs (n = 3). C) Western blot analysis of PD‐1 from HSCs and HSCs‐PD‐1 (n = 3). GAPDH served as an internal reference protein. D) Fluorescence images of HSCs and HSCs‐PD‐1. Cell nuclei were stained with DAPI (blue), while the cytomembrane was stained with Alexa Fluor 594‐conjugated wheat germ agglutinin (WGA594) (red). Green refers to the EGFP signal. Scale bar: 5 µm. E) The interaction of HSCs‐PD‐1 and PD‐L1‐expressing B16F10 melanoma cells was observed using confocal microscopy. Representative fluorescence imaging and quantitative analysis of HSCs and HSCs‐PD‐1, which were stained with DAPI (blue) and PE‐CD44 antibody (orange). CD44 is a marker of HSCs. Scale bar: 20 µm. F–H) Representative flow cytometry images and quantitative analysis of key markers of HSCs‐PD‐1 and SB@HSCs‐PD‐1 under the same voltage, including Sca‐1 (F), CXCR4 (G), and PD‐1 (H) (n = 3). I) In vivo bone‐targeting effect of HSCs‐PD‐1 and SB@HSCs‐PD‐1, as assessed using IVIS images. Cells were labeled with DiD (n = 4). J) Representative flow cytometric image of EGFP^+^ cells in the bone marrow, on the 0th, 1st, and 8th days after treatment with EGFP^+^ HSCs. K) Quantitative analysis of EGFP^+^ cells in major organs, on the 0th, 1st, and 8th days after treatment with EGFP^+^ HSCs (n = 3). Data have been represented as mean ± SD. Statistical significance was calculated using Student's *t*‐test and one‐way ANOVA followed by Tukey's post‐hoc test; *P*‐value: *P<0.05, **P<0.01, and ****P<0.0001; n.s., no significance; a.u., arbitrary units; MFI, mean fluorescence intensity.

To further take advantage of HSCs as targeting platforms for metastatic bone and improve therapeutic outcomes, we generated genetically engineered HSCs by means of transfection with lentivirus‐EGFP‐PD‐1 vector (HSCs‐PD‐1). Similar to the anti‐PD‐L1 antibody, HSCs‐PD‐1 can serve as a trap to bind PD‐L1^+^ cells at metastatic bone sites, which is an alternative genetic strategy for ICB antibodies (**Figure** **5**A). Flow cytometry and western blot analysis of PD‐1 on HSCs were performed to study the transfection efficiency of viruses with different titers, which suggested that five multiplicities of infection of the virus displayed effective transfection efficiency (Figure 5B,C and Figure S6A, Supporting Information). In addition, fluorescence images revealed an enhanced green fluorescent protein (EGFP) signal in HSCs‐PD‐1, indicating that the lentivirus‐EGFP‐PD‐1 vector was successfully transfected into HSCs (Figure 5D). We obtained stable PD‐1‐transfected stem cells within five days of puromycin screening (Figure S6B, Supporting Information). We found that the level of PD‐L1 expression in B16F10 cells in bone metastases was higher than that in B16F10 cells cultured in vitro (Figure S7A, Supporting Information), which may be due to the microenvironment within bone metastases promoting tumor cells to overexpress PD‐L1.^[^ ^27^ ^]^ To test the binding ability of HSCs‐PD‐1 to PD‐L1 in tumors, we used lentiviral transfection to overexpress PD‐L1 in B16F10 cells in vitro (Figure S7B,C, Supporting Information) and then co‐cultured HSCs‐PD‐1 or HSCs with adherent B16F10‐PD‐L1 cells for 3 h. The unbound supernatant cells were removed by washing with PBS. The adherent cells were stained with 4',6‐diamidino‐2‐phenylindole (DAPI) and PE‐conjugated anti‐CD44 antibodies, the latter of which is a marker of HSCs. Compared to cancer cells co‐cultured with HSCs, HSCs‐PD‐1 co‐incubation displayed a dramatic increase in CD44 signaling. This result suggested that HSCs‐PD‐1 could adhere to B16F10‐PD‐L1 cells via PD‐1/PD‐L1 binding (Figure 5E). Meanwhile, we confirmed that the expression levels of Sca‐1, CXCR4, PD‐1, c‐kit, and CD44, as well as the bone‐homing capacity of HSCs‐PD‐1, did not change significantly after SB loading (Figure 5F–I and Figure S7D,E, Supporting Information).

More intriguingly, we observed that HSCs‐PD‐1 were able to reduplicate themselves within the bone marrow niche after administration. Myeloid EDFP^+^ cells increased from 0.002% to 0.057% within 8 d, indicating that as “living drugs”, HSCs‐PD‐1 can proliferate within the bone marrow and continuously express PD‐1, to block PD‐L1^+^ cells (Figure 5J,K). In addition, we compared the proportion of EGFP^+^ cells in the bone tissue and other major organs on the 1st and 8th days. It is worth noting that EGFP signals decreased in other accumulation tissues, such as the liver and lungs (Figure 5K and Figure S8, Supporting Information). This is likely due to the specific niches within the bone marrow, which determine the proliferative status.^[^ ^28^ ^]^ As a result, more PD‐1 molecules are expressed in the metastatic bone marrow, to avoid PD‐L1‐induced PD‐1^+^CD8^+^ T cell dysfunction and exhaustion.

### 2.6 Anti‐Tumor Immunotherapy using SB@HSCs‐PD‐1

**Figure 6:**
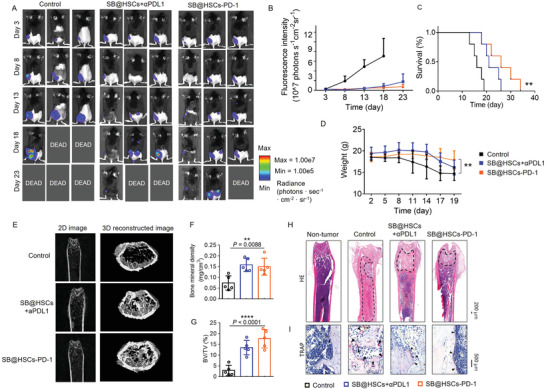
Therapeutic efficacy of SB@HSCs‐PD‐1 in bone metastases. A,B) The growth of bone metastases was monitored using IVIS. Representative IVIS images (A) and quantification (B) of tumor growth in each group, including control (n = 5), SB@HSCs+*α*PD‐L1 (n = 5), and SB@HSCs‐PD‐1 (n = 5). C) Survival curve of mice after different treatments. D) Body weight of mice in the different treatment groups. E) Representative micro‐CT 2D images and 3D reconstructed images of the distal part of the tumor‐bearing femurs. F,G) Quantitative analysis of bone destruction in the different groups, including BMD (F) and BV/TV (G) (n = 5). H,I) Histological images for H&E (H) and TRAP staining (I) of femurs from mice, after different treatments. The tumor sites are circled using a dotted line. Data in Figure 6B have been represented as mean ± SEM, while data in the other figures have been represented as mean ± SD. Statistical significance was calculated using one‐way ANOVA followed by Tukey's post‐hoc test, *P*‐value: **P<0.01 and ****P<0.0001. Asterisks in Figure 6C indicate the statistical significance compared to the control, as analyzed using log‐rank (Mantel–Cox) test; *P*‐value: **P<0.01.

To evaluate the therapeutic efficacy of SB@HSCs‐PD‐1 in vivo, bone metastases models were left untreated or intravenously infused with SB@HSCs‐PD‐1 (1×10^5^ cells/mouse) every three days, for four administrations. The combination treatment of the same dosing frequency of SB@HSCs (1×10^5^ cells/mouse) and anti‐PD‐L1 (40 µg/mouse) was used as a positive control. Based on the bioluminescence images and quantitative analysis, bone metastases displayed significant regression following SB@HSCs‐PD‐1 administration, which afforded better therapeutic outcomes than the combination treatment (**Figure** **6**A,B and Figure S9, Supporting Information). In addition, SB@HSCs‐PD‐1 treatment significantly lengthened the survival of mice, as compared to that of mice that received SB@HSCs with anti‐PD‐L1 mAbs (Figure 6C). Furthermore, the weight of the untreated mice decreased significantly, but the weight loss of the mice treated with SB@HSCs‐PD‐1 was significantly lower than that of the controls, indicating better therapeutic efficacy of SB@HSCs‐PD‐1 in controlling bone metastasis (Figure 6D). This better therapeutic outcome may predominantly result from the self‐replication of HSCs‐PD‐1 within the bone marrow, which does not happen in the case of anti‐PD‐L1 mAbs.

To assess the effect of SB@HSCs‐PD‐1 on the morphological structures of bone, we also performed micro‐CT analysis with 2D and 3D reconstructed images ex vivo, on the distal part of the femur. Mice treated with SB@HSCs‐PD‐1 showed less bone destruction than those in the control group (Figure 6E). In addition, bone histomorphometric parameters, including BMD and BV/TV, were obviously increased in mice receiving SB@HSCs‐PD‐1, suggesting that tumor regression inhibited bone destruction (Figure 6F,G). We further observed the structure of bone metastases in the bone marrow using hematoxylin and eosin (H&E) staining. Bone metastases after SB@HSCs‐PD‐1 treatment were significantly inhibited compared to those in the control group (Figure 6H). Tartrate‐resistant acid phosphatase (TRAP) staining revealed that the number of osteoclasts had decreased after treatment (Figure 6I). These results implied that SB@HSCs‐PD‐1 reduced bone destruction.

**Figure 7:**
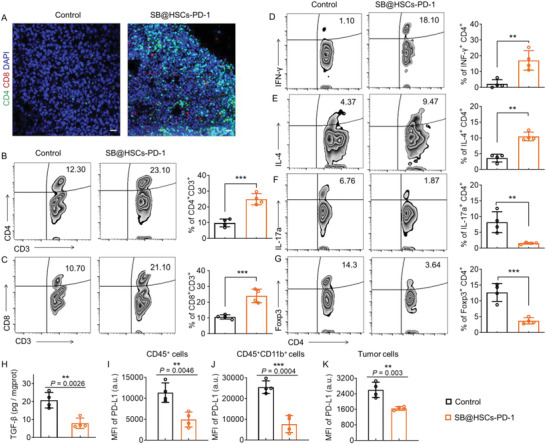
A) Representative immunofluorescence images of CD4^+^ (green) and CD8^+^ (red) T cells in bone metastases, after different treatments. Scale bar: 20 µm. B–G) Representative flow cytometry zebra plots and statistical analyses of immune cells in mice, after different treatments. CD4^+^ T cells (B), CD8^+^ T cells (C), T_H_1 (D), T_H_2 (E), T_H_17 (F), and T_reg_ (G) cells (n = 4). H) TGF‐*β* levels in the bone marrow of mice in each group (n = 4). I–K) PD‐L1 expression levels of CD45^+^ (I), CD45^+^ CD11b^+^ (J), and tumor (K) cells in all groups, as analyzed using flow cytometry (n = 4). Data have been represented as mean ± SD. Statistical significance was calculated using a two‐tailed unpaired Student's *t*‐test (n = 4); *P*‐value: *P<0.05, **P<0.01, and ***P<0.001; a.u., arbitrary units; MFI, mean fluorescence intensity.

We investigated the mechanism of action of SB@HSCs‐PD‐1 therapy in bone metastases. Compared to untreated mice, there was a sharp increase in the infiltration of CD4^+^ and CD8^+^ T cells in the bone metastatic tumors in mice that received SB@HSCs‐PD‐1 (**Figure** **7**A), as observed using immunofluorescence imaging. Next, we performed flow cytometry analysis of the tumor‐infiltrating immune cells collected from the bone metastasis marrow. Consistent with the confocal results, a marked increase in the proportions of CD4^+^ and CD8^+^ T cells was observed in the mice treated with SB@HSCs‐PD‐1, suggesting activation of infiltrated T cells (Figure 7B,C). Compared with control group, the PD‐1 level of CD4^+^ T cells in mice with SB@HSCs‐PD‐1 treatment did not change significantly (Figure S10A, Supporting Information), but increased in CD8^+^ T cells (Figure S10B, Supporting Information), which implied that bone metastases shifted to immunologically “hot” phenotype after SB@HSCs‐PD‐1 treatment.^[^ ^22^ ^]^ The main populations of CD4^+^ T cells infiltrating the bone metastasis encompassed T_H_1, T_H_2, T_H_17, and T_reg_ cells. We found that SB@HSCs‐PD‐1 treatment amplified the ratios of T_H_1 and T_H_2 cells, while concomitantly reducing the proportions of T_H_17 and T_reg_ cells (Figure 7D–G). We also found that the level of TGF‐*β* in the bone marrow was significantly reduced compared to that in the control group after SB@HSCs‐PD‐1 treatment (Figure 7H). More importantly, there were significantly decreased PD‐L1 levels in CD45^+^ and CD45^+^ CD11b^+^ cells, as well as tumor cells, indicating that our HSCs platform could effectively express PD‐1 for a long time, to block PD‐L1 (Figure 7I–K). Meanwhile, the systemic level of IL‐6 in the serum was not significantly elevated, indicating that SB@HSCs‐PD‐1 therapy was tolerated in mice (Figure S10C, Supporting Information). H&E staining of other major tissues confirmed that SB@HSCs‐PD‐1 had little toxicity in the treated mice (Figure S10D, Supporting Information).

## 3 Conclusion

In this study, we delineated an alternative cell engineering strategy, ICB, to target bone metastasis, using HSCs as a platform. Compared with “dead drugs” such as PD‐L1 mAbs, the HSCs‐PD‐1 as “living drugs” can amplify themselves through cell proliferation when they access the bone marrow. They can express PD‐1 molecules for a long time in bone metastasis therapy. In addition, the side effects and irAEs can also be reduced in other normal organs as these “living drugs” showed little ability to proliferate in the other organs except the bone marrow. Successful reversal of the immunosuppressive microenvironment in bone metastatic tumors was realized by SB@HSCs‐PD‐1, thereby effectively reactivating anti‐tumor immunity in this immune‐privileged area. The homing effect of HSCs is mediated by complex intermolecular interactions between the HSCs and the bone microenvironment. This technology may also inspire the expression of other therapeutic proteins in HSCs for bone disease treatment. Owing to the ease of preparation, plasticity, feasibility, and biosafety of the HSCs platform, this novel technology may have a promising clinical translation potential.

## 4 Experimental Section

### Cell Lines

Luciferase‐labeled B16F10 melanoma cells (B16F10‐Luc) were obtained from Prof. Z. Liu at the Soochow University, Suzhou, Jiangsu, China. The cells were cultured in Dulbecco's modified Eagle medium supplemented with 10% fetal bovine serum and 1% penicillin‐streptomycin, and placed in a 5% CO_2_‐containing atmosphere, at 37 °C.

### Mice

C57BL/6 mice (6–8 weeks, female) were purchased from Changzhou Cavens Experimental Animal Co. Ltd, Changzhou, Jiangsu, China. The experimental group sizes were approved by the regulatory authorities for animal welfare, after being defined to balance statistical power, feasibility, and ethical aspects. All animal experiments were approved by the Institutional Animal Care and Use Committee of Soochow University (SUDA20211025A04).

### Establishment of Bone Metastasis Model

A bone metastasis model was established as previously described.^[^ ^29^ ^]^ The mice were anesthetized with isoflurane. After depilation of the left leg, the patellar ligament was exposed and the insulin needle was carefully inserted at the site of the intercondylar notch of the left femur into the femoral cavity. Following smooth penetration, 25 µL B16F10‐Luc cell suspension (1×10^6^ cells) was slowly injected into the left femur. The needle was then extracted, and the injection site was sealed with bone wax (Ethicon, Cincinnati, Ohio, USA), to prevent leakage of tumor cells. Finally, the left leg was sutured and the mice were given suitable post‐surgical care.

### Exploration of the Immune Microenvironment in Bone Metastases

Flow cytometry was used to evaluate the immune microenvironment of bone metastases. Since there were no mature CD4^+^ T cells in healthy bone marrow, subsets of CD4^+^ T cells were compared from bone metastases and subcutaneous tumors (Figure S1D, Supporting Information). Fluorochrome‐conjugated antibodies against mouse CD45, CD11b, CD11c, PD‐L1, CD3, CD8, CD4, PD‐1, IFN‐*γ*, IL‐17a, IL‐4, Tim‐3, Lag‐3, and Foxp3 were purchased from BioLegend (San Diego, California, USA). Following euthanasia of the mice, bone marrow and tumor cells were extracted from them, by flushing of the femurs. Single‐cell suspensions were filtered through a 300‐mesh nylon gauze and treated with RBC lysis buffer (Solarbio, Beijing, China). Cell surface staining was conducted by incubating the cells with antibodies for 1.5 h, in flow cytometry buffer (3% bovine serum albumin in PBS). For intracellular staining, the cells were fixed and incubated with antibodies in the IntraPrep permeabilization reagent for 40 min. The stained cells were analyzed using a BD Accuri C6 flow cytometer (Franklin Lakes, New Jersey, USA).

TGF‐*β* levels were determined using an enzyme‐linked immune sorbent assay (ELISA) kit. Bone metastatic and subcutaneous tumor tissues were collected separately and lysed with tissue lysates for 4 h, after crushing. The samples were centrifuged at 2000 rpm for 15 min, following which the supernatants were collected. The concentration of TGF‐*β* in different tumors was detected according to the protocol of the Mouse TGF‐*β*1 Pre‐coated ELISA Kit (Dakewe Biotech Co. Ltd., Shenzhen, Guangzhou, China). The total protein concentration in the tumor was also evaluated using a BCA kit (Beyotime Biotechnology, Shanghai, China). The relative TGF‐*β* level in the tumor was calculated as TGF‐*β*/total protein concentration.

### Extraction and Cultivation of HSCs

HSCs were collected according to the manufacturer's protocol for the magnetic bead‐based EasySep Mouse SCA1 Positive Selection Kit (StemCell Technologies, Vancouver, British Columbia, Canada). Briefly, bone cells were collected from the femur and tibia of C57BL/6 mice and made into a suspension of 1×10^6^ cells mL^−1^ in StemSpan Serum‐Free Expansion Medium (SFEM) (StemCell Technologies) containing human IL‐6 (100 ng mL^−1^, StemCell Technologies, Canada), human fms‐like tyrosine kinase‐3 (Flt3) ligand (100 ng mL^−1^, StemCell Technologies), murine stem‐cell factor (SCF) (50 ng mL^−1^, StemCell Technologies), and human thrombopoietin (TPO) (20 ng mL^−1^, PeproTech, Cranbury, New Jersey, USA). Mouse SCA1 PE Labeling Reagent (50 µL mL^−1^, StemCell Technologies) was first added to the cell suspension, and the mixture was kept in the dark at room temperature for 15 min. Then, a PE Selection Cocktail (70 µL mL^−1^, StemCell Technologies) was added to the mixture and kept in the dark at room temperature for 15 min. After vortexing, dextran RapidSphere (50 µL mL^−1^, StemCell Technologies) was added to the mixture and stored at room temperature in the dark for 10 min. After the mixture was incubated in the magnet for 5 min, the supernatant was discarded and SFEM was added to resuspend the cells. This step was repeated three times to obtain HSCs, and the cells were used in passages 3–5.

### Characterization of HSCs

The purity of the HSCs was analyzed using flow cytometry, after staining with antibodies against the HSC‐specific markers Sca‐1 and CXCR4. The structure of the HSCs was analyzed by means of fluorescence staining with DAPI (Beyotime Biotechnology) and Alexa Fluor 594‐conjugated wheat germ agglutinin (Vector Laboratories, Burlingame, California, USA), in the dark at room temperature for 30 min. The cells were then washed with PBS, suspended in 500 µL PBS, and observed under a confocal microscope (ZEISS LSM 800 with Airyscan, Oberkochen, Baden‐Württemberg, Germany).

### Preparation and Characterization of SB‐Loaded HSCs (SB@HSCs)

Different concentrations of the TGF‐*β* inhibitor SB‐505124 (SB, MedChemExpress, New Jersey, USA) were added to the HSC suspension (5–50 µg mL^−1^), and these mixtures were oscillated gently for 2 h at 4 °C. After centrifugation, HSCs, SB, and SB@HSCs were scanned using a UV–vis spectrophotometer (Lambda 750, PerkinElmer, Waltham, Massachusetts, USA), to verify the load of SB.

In addition, the loading amount, loading percentage, and released drug rate of SB@HSCs were determined using HPLC (UltiMate 3000, ThermoFisher, Waltham, Massachusetts, USA). Loading percentage (%) = weight of SB in SB@HSCs / initial amount of SB × 100. SB@HSCs containing 7.5 µg SB (7.5 µg/1×10^5^ HSCs) were obtained, at a concentration of 50 µg mL^−1^ SB, following which the SB release rate was determined according to published literature.^[^ ^30^ ^]^ Briefly, 1 mL PBS was added to the wells of a 12‐well plate equipped with a 3 µm Transwell, and SB@HSCs were placed in the upper chamber. The plate was stored in an incubator maintained at 37 °C, at a shaking rate of 100 rpm. At specified time‐points, 1 mL of release medium was replaced with the same volume of fresh PBS. SB concentration was determined using HPLC, and the UV detection wavelength used was 300 nm. It was confirmed that the release experiment fulfilled the sink condition.

After SB loading, the expression of HSCs surface proteins was assessed using flow cytometry. HSCs and SB@HSCs were stained with PE‐Sca‐1, APC‐CXCR4, FITC‐c‐kit, and PE‐CD44, and the stained cells were analyzed using a BD Accuri C6 flow cytometer.

### In Vivo Targeting to Bone

Purified HSCs were incubated in DiD (Jiangsu KeyGEN BioTECH Corp. Ltd., Nanjing, Jiangsu, China) containing PBS for 30 min, to obtain DiD‐labeled cells. One portion of the DiD‐labeled HSCs was treated with 3.7% paraformaldehyde as a control. DiD‐labeled HSCs and paraformaldehyde‐fixed DiD‐HSCs were intravenously injected into the mice. Four hours later, the mice were sacrificed, and their hind limb bones were isolated for fluorescence imaging using an IVIS Lumina III In Vivo Imaging System (PerkinElmer). The exposure time was 20 s.

To further investigate the bone‐targeting efficiency of SB@HSCs, free DiD and SB@HSCs/DiD were injected into the tail vein. After different time points, the mice were sacrificed, and their major organs and hind limb bones were isolated for fluorescence imaging using IVIS. The exposure time was set to 3 s. And the exposure time for shooting individual bones was set to 20 s.

### Cytotoxicity Assay

HSCs (1×10^4^ cells well^−1^) were seeded into a 96‐well plate. At the specified time‐points, SB was added to the cell suspensions, at different concentrations. After 24 h, 3‐(4,5‐Dimethylthiazol‐2‐yl)‐2,5‐diphenyltetrazolium bromide (MTT) reagent was added, and the cells were incubated for another 4 h. The supernatant was then removed, and the residue was dissolved in dimethyl sulfoxide. The absorbance of the solution was measured at the wavelength of 570 nm using a microplate reader (Synergy H1, Bio‐tek, Vermont, USA).

The concentration of SB in the intravenously injected SB@HSCs was 37.5 µg mL^−1^. The concentration of SB when preparing SB@HSCs was 50 µg mL^−1^. Therefore, the Apoptosis and Necrosis Assay Kit (Beyotime Biotechnology) was also used to detect the toxicity of SB towards HSCs, at SB concentrations of 50 and 37.5 µg mL^−1^, respectively.

### Therapeutic Treatment

Bone metastases models were left untreated or intravenously injected with HSCs (1×10^5^ cells/mouse), SB (7.5 µg/mouse), anti‐PD‐L1 (40 µg/mouse), SB@HSCs (1×10^5^ cells/mouse), or a combination of SB@HSCs (1×10^5^ cells/mouse) and anti‐PD‐L1 (40 µg) in 0.2 mL PBS every three days, for four administrations. After treatment, the immune microenvironment in bone metastases was evaluated using flow cytometry on the 17th day. The antibodies used for flow cytometric analysis were the same as those used to explore the immune microenvironment in bone metastases.

### Bioluminescence Imaging

After the mice were anesthetized, D‐luciferin (10 µL g^−1^, PerkinElmer) was intraperitoneally injected into them. After 10 min, the mice were imaged using an IVIS Lumina III In Vivo Imaging System. The exposure time for each mouse was 1 min.

### Immunofluorescence Staining

Tumor tissues were frozen in O.C.T. compound (Solarbio) and cryosectioned to a thickness of 10 µm using a cryotome. These sections were then fixed in 4% paraformaldehyde solution for 30 min, washed twice with PBS, blocked with FACS buffer for 30 min, and stained with fluorescein isothiocyanate (FITC)‐conjugated anti‐CD4 and PE‐conjugated anti‐CD8 antibodies, overnight in the dark at 4 °C. The next day, the sections were stained with DAPI. After washing twice with PBS containing 0.1% Tween 20, the sections were observed using confocal microscopy.

### Micro‐CT

Femurs were collected and fixed in 4% paraformaldehyde solution. A SkyScan 1176 µCT scanner (SkyScan, Aartselaar, Belgium) was used to detect bone destruction. The scanning parameters were as follows: voltage, 50 kV; current, 810 mA; resolution, 10 µm. The 3D model of the cortical and cancellous bones of the distal femur was reconstructed using Mimics software (version 16.0; Materialise Corp., Leuven, Belgium). The BMD and BV/TV (%) of the samples were measured.

### Bone Histology

The fixed femurs were demineralized by decalcifying with 10% EDTA (Solarbio) for one week. The samples were then dehydrated using gradient alcohol solutions (75%, 85%, 90%, 95%, and 100%) and embedded in paraffin. Sections for trabecular bone were obtained from the distal femur, at a thickness of 5 µm, using a microtome for H&E and TRAP staining.

### Preparation and Characterization of HSCs‐PD‐1 and SB@HSCs‐PD‐1

To prepare genetically engineered HSCs, a lentivirus vector (pRLenti‐EF1‐EGFP‐P2A‐Puro‐CMV‐Pdcd1‐3×FLAG‐WPRE) purchased from OBiO Technology Corp. Ltd. (Shanghai, China) was cultured with HSCs, at 37 °C for 48 h. Stable strains were screened with 5 µg mL^−1^ puromycin, to obtain HSCs‐PD‐1.

The expression of PD‐1 was confirmed using flow cytometry, western blot, and confocal microscopy. HSCs‐PD‐1 were stained with APC‐PD‐1 antibody for 1.5 h and analyzed using a BD Accuri C6 flow cytometer, after centrifugation. According to the western blot protocol, HSCs‐PD‐1 were lysed with radioimmunoprecipitation assay lysis buffer, at 4 °C for 40 min. After centrifugation at 2000 rpm for 15 min, the extracted protein was added to the loading buffer and boiled, separated by means of 10% sodium dodecyl sulfate‐polyacrylamine gel electrophoresis, and transferred to a polyvinylidene fluoride (PVDF) membrane. The PVDF membrane was then blocked with FACS buffer, incubated with anti‐PD‐1 (1:1000) and anti‐GAPDH (1:1000) antibodies, overnight at 4 °C, and further detected using horseradish peroxidase‐conjugated goat anti‐rabbit IgG secondary antibody (1:5000). FluoChemr (ProteinSimple, San Francisco, California, USA) was used to display the hybridization bands, while ImageJ software was used for quantitative analysis. The structure of HSCs‐PD‐1 was observed by means of fluorescence staining with DAPI and Alexa Fluor 594‐conjugated wheat germ agglutinin, in the dark at room temperature for 30 min. After washing with PBS, the cells were suspended in 500 µL of PBS and observed under a confocal microscope. The expression of PD‐1 in the cells was further detected using ELISA. HSCs‐PD‐1 (1×10^5^) were collected and lysed with cell lysate on ice for 4 h. After centrifugation, the concentration of PD‐1 in the supernatant was determined using the Mouse PD‐1 ELISA Kit (Beyotime Biotechnology), according to the manufacturer's instructions. Finally, the expression of PD‐1 in each HSCs‐PD‐1 cell was found to be approximately 1.271×10^−9^ ± 2.942 ×10^−10^ µmol.

Moreover, SB (50 µg mL^−1^) was mixed with the HSCs‐PD‐1 suspension for 4 h and then centrifuged at 500 × *g* for 3 min, following which the supernatant was discarded to obtain SB@HSCs‐PD‐1. After SB loading, the expression of key HSCs‐PD‐1 surface proteins was assessed using flow cytometry, with antibodies against PerCP‐PD‐1, PE‐Sca‐1, and APC‐CXCR4.

To test the binding ability of HSCs‐PD‐1 to PD‐L1, a lentivirus (HBLV‐m‐CD274‐3×flag‐ZsGreen‐PURO, Hanbio Biotechnology Co. Ltd., Shanghai, China) was used to transfect B16F10 cells and overexpress PD‐L1. B16F10‐PD‐L1 cells were co‐cultured with HSCs‐PD‐1 or HSCs for 3 h. The unbound supernatant cells were removed by washing with PBS. The adherent cell mixture was then washed and stained with DAPI and PE‐conjugated anti‐CD44 antibodies, which were markers of HSCs, following which the number of bound HSCs was observed using fluorescence microscopy.

In addition, the survival and proliferation of SB@HSCs‐PD‐1 in vivo were verified by detecting EGFP signals using flow cytometry. Bone marrow cells were collected on days 0, 1, and 8, to explore the changes in the content of EGFP^+^ cells in the bone marrow. The content of EGFP^+^ cells in other organs was also measured, on the 1st and 8th days.

### Therapeutic Effect of SB@HSCs‐PD‐1

Mice with bone metastases were intravenously injected with PBS, SB@HSCs‐PD‐1 (1×10^5^ cells/mouse), or a combination of SB@HSCs (1×10^5^ cells/mouse) with anti‐PD‐L1 (40 µg) in 0.2 mL PBS every three days, for four administrations. The tumor growth rate was monitored using an IVIS Lumina III In Vivo Imaging System, with an exposure time of 1 min. The body weight and survival of the mice were also recorded. On the 18th day, three mice from each group were injected with 10 µL g^−1^ D‐luciferin. Ten minutes later, the mice were euthanized and dissected, and the secondary metastasis of bone metastases was explored using an IVIS Lumina III In Vivo Imaging System. The exposure time was 1 min. After treatment, changes in the immune microenvironment of bone metastases in the control and SB@HSC‐PD‐1 groups were characterized using flow cytometry, as described above.

### In Vivo Safety Test of SB@HSCs‐PD‐1

Blood samples from the control and SB@HSCs‐PD‐1 groups were collected on the 5th, 10th, and 15th days. After blood clotting at 4 °C, the samples were centrifuged at 2000 × *g* for 10 min, following which the serum was collected. A mouse IL‐6 uncoated ELISA kit (Invitrogen, Carlsbad, California, USA) was used to detect the level of IL‐6 in the serum.

To investigate the safety of SB@HSCs‐PD‐1, the major organs of mice treated with SB@HSCs‐PD‐1 were isolated and fixed in 4% paraformaldehyde. Samples embedded routinely in paraffin were cut into 4 µm slices and stained with H&E. Finally, the samples were observed under a DM4000 microscope (Leica, Wetzlar, Germany).

### Statistical Analysis

All data have been expressed as mean ± SD, unless otherwise specified. Data were obtained from at least three independent measurements (*n*≥3). Statistical analyses were performed using Prism v6.0 (GraphPad, San Diego, California, USA) with the appropriate tests. The significance of the differences between two groups was calculated using a two‐tailed unpaired Student's *t*‐test. Analysis of variance and Tukey's post‐hoc test was performed between more than two groups. *P* values have been indicated as *P<0.05, **P<0.01, ***P<0.001, and ****P<0.0001.

## Conflict of Interest

The authors declare no conflict of interest.

## Author Contributions

C.W., X.Z., and J.B. designed the project. B.W., J.B., B.T., and H.C. performed the experiments and collected the data. B.W. and J.B. analyzed and interpreted the data. All authors contributed to the writing of the manuscript, discussed the results and implications, and edited the manuscript at all stages.

## Supporting information

Supporting Information

## Data Availability

The data that support the findings of this study are available from the corresponding author upon reasonable request.

## References

[1] a) N. Kamiya , H. Suzuki , T. Endo , M. Yano , M. Naoi , D. Nishimi , K. Kawamura , T. Imamoto , T. Ichikawa , Int. J. Urol. 2012, 19, 968; 22805007 10.1111/j.1442-2042.2012.03098.x

[2] E. F. Solomayer , I. J. Diel , G. C. Meyberg , C. Gollan , G. Bastert , Breast Cancer Res. Treat. 2000, 59, 271. 10832597 10.1023/a:1006308619659

[3] P. Sharma , R. K. Pachynski , V. Narayan , A. Flechon , G. Gravis , M. D. Galsky , H. Mahammedi , A. Patnaik , S. K. Subudhi , M. Ciprotti , T. Duan , A. Saci , S. Hu , G. C. Han , K. Fizazi , J. Clin. Oncol. 2019, 37, 142.

[4] R. E. Coleman , Clin. Cancer Res. 2006, 12, 6243s. 17062708 10.1158/1078-0432.CCR-06-0931

[5] a) K. Wang , Y. Gu , Y. Liao , S. Bang , C. R. Donnelly , O. Chen , X. Tao , A. J. Mirando , M. J. Hilton , R. R. Ji , J. Clin. Invest. 2020, 130, 3603; 32484460 10.1172/JCI133334 PMC7324182

[6] a) K. M. Hargadon , C. E. Johnson , C. J. Williams , Int. Immunopharmacol. 2018, 62, 29; 29990692 10.1016/j.intimp.2018.06.001

[7] S. Jiao , S. K. Subudhi , A. Aparicio , Z. Ge , B. Guan , Y. Miura , P. Sharma , Cell 2019, 179, 1177. 31730856 10.1016/j.cell.2019.10.029

[8] C. Liu , M. Wang , C. Xu , B. Li , J. Chen , J. Chen , Z. Wang , J. Immunol. Res. 2021, 2021, 8970173. 34877360 10.1155/2021/8970173 PMC8645368

[9] a) T. M. Beer , E. D. Kwon , C. G. Drake , K. Fizazi , C. Logothetis , G. Gravis , V. Ganju , J. Polikoff , F. Saad , P. Humanski , J. M. Piulats , P. Gonzalez Mella , S. S. Ng , D. Jaeger , F. X. Parnis , F. A. Franke , J. Puente , R. Carvajal , L. Sengelov , M. B. McHenry , A. Varma , A. J. van den Eertwegh , W. Gerritsen , J. Clin. Oncol. 2017, 35, 40; 28034081 10.1200/JCO.2016.69.1584

[10] T. Trivedi , G. M. Pagnotti , T. A. Guise , K. S. Mohammad , Biomolecules 2021, 11, 1643. 34827641 10.3390/biom11111643 PMC8615596

[11] B. Zhang , Y. Li , Q. Wu , L. Xie , B. Barwick , C. Fu , X. Li , D. Wu , S. Xia , J. Chen , W. P. Qian , L. Yang , A. O. Osunkoya , L. Boise , P. M. Vertino , Y. Zhao , M. Li , H. R. Chen , J. Kowalski , O. Kucuk , W. Zhou , J. T. Dong , Nat. Commun. 2021, 12, 1714. 33731701 10.1038/s41467-021-21976-w PMC7969754

[12] J. Seoane , R. R. Gomis , Cold Spring Harbor Perspect. Biol. 2017, 9, a022277. 10.1101/cshperspect.a022277 PMC5710110 28246180

[13] L. I. Gold , Crit. Rev. Oncog. 1999, 10, 303. 10654929

[14] a) M. Saeed , F. Chen , J. Ye , Y. Shi , T. Lammers , B. G. De Geest , Z. P. Xu , H. Yu , Adv. Mater. 2021, 33, 2008094; 10.1002/adma.202008094 34048101

[15] P. Juarez , K. S. Mohammad , J. J. Yin , P. G. Fournier , R. C. McKenna , H. W. Davis , X. H. Peng , M. Niewolna , D. Javelaud , J. M. Chirgwin , A. Mauviel , T. A. Guise , Cancer Res. 2012, 72, 6247. 23002206 10.1158/0008-5472.CAN-12-1444 PMC4447239

[16] S. Herbertz , J. S. Sawyer , A. J. Stauber , I. Gueorguieva , K. E. Driscoll , S. T. Estrem , A. L. Cleverly , D. Desaiah , S. C. Guba , K. A. Benhadji , C. A. Slapak , M. M. Lahn , Drug Des., Dev. Ther. 2015, 9, 4479. 10.2147/DDDT.S86621 PMC4539082 26309397

[17] Y. Lan , D. Zhang , C. Xu , K. W. Hance , B. Marelli , J. Qi , H. Yu , G. Qin , A. Sircar , V. M. Hernandez , M. H. Jenkins , R. E. Fontana , A. Deshpande , G. Locke , H. Sabzevari , L. Radvanyi , K.‐M. Lo , Sci. Transl. Med. 2018, 10, eaan5488. 29343622 10.1126/scitranslmed.aan5488

[18] J. Strauss , C. R. Heery , J. Schlom , R. A. Madan , L. Cao , Z. Kang , E. Lamping , J. L. Marte , R. N. Donahue , I. Grenga , L. Cordes , O. Christensen , L. Mahnke , C. Helwig , J. L. Gulley , Clin. Cancer Res. 2018, 24, 1287. 29298798 10.1158/1078-0432.CCR-17-2653 PMC7985967

[19] L. Paz‐Ares , T. M. Kim , D. Vicente , E. Felip , D. H. Lee , K. H. Lee , C. C. Lin , M. J. Flor , M. Di Nicola , R. M. Alvarez , I. Dussault , C. Helwig , L. S. Ojalvo , J. L. Gulley , B. C. Cho , J. Thorac. Oncol. 2020, 15, 1210. 32173464 10.1016/j.jtho.2020.03.003 PMC8210474

[20] a) T. Li , H. Dong , C. Zhang , R. Mo , Nano Res. 2018, 11, 5240;

[21] a) Q. Ma , Q. Fan , J. Xu , J. Bai , X. Han , Z. Dong , X. Zhou , Z. Liu , Z. Gu , C. Wang , Matter 2020, 3, 287; 32835220 10.1016/j.matt.2020.05.017 PMC7242942

[22] M. Binnewies , E. W. Roberts , K. Kersten , V. Chan , D. F. Fearon , M. Merad , L. M. Coussens , D. I. Gabrilovich , S. Ostrand‐Rosenberg , C. C. Hedrick , R. H. Vonderheide , M. J. Pittet , R. K. Jain , W. Zou , T. K. Howcroft , E. C. Woodhouse , R. A. Weinberg , M. F. Krummel , Nat. Med. 2018, 24, 541. 29686425 10.1038/s41591-018-0014-x PMC5998822

[23] M. Liu , F. S. Kuo , K. J. Capistrano , D. A. Kang , B. G. Nixon , W. Shi , C. Chou , M. H. Do , E. G. Stamatiades , S. Y. Gao , S. Li , Y. B. Chen , J. J. Hsieh , A. A. Hakimi , I. Taniuchi , T. A. Chan , M. O. Li , Nature 2020, 587, 115. 33087928 10.1038/s41586-020-2836-1 PMC8347705

[24] Y. Taniguchi , K. Kawano , T. Minowa , T. Sugino , Y. Shimojo , Y. Maitani , Cancer Sci. 2010, 101, 2207. 20608940 10.1111/j.1349-7006.2010.01646.x PMC11159942

[25] a) P. I. Croucher , M. M. McDonald , T. J. Martin , Nat. Rev. Cancer 2016, 16, 373; 27220481 10.1038/nrc.2016.44

[26] A. E. Chiou , C. Liu , I. Moreno‐Jimenez , T. T. Tang , W. Wagermaier , M. N. Dean , C. Fischbach , P. Fratzl , Sci. Adv. 2021, 7, eabf2283. 33731354 10.1126/sciadv.abf2283 PMC7968847

[27] I. Vitale , E. Shema , S. Loi , L. Galluzzi , Nat. Med. 2021, 27, 212. 33574607 10.1038/s41591-021-01233-9

[28] a) A. Merchant , G. Joseph , W. Matsui , Blood 2008, 112, 1390; 10.1182/blood-2009-09-241703 PMC2845897 20107231

[29] W. Zhang , I. L. Bado , J. Hu , Y. W. Wan , L. Wu , H. Wang , Y. Gao , H. H. Jeong , Z. Xu , X. Hao , B. M. Lege , R. Al‐Ouran , L. Li , J. Li , L. Yu , S. Singh , H. C. Lo , M. Niu , J. Liu , W. Jiang , Y. Li , S. T. C. Wong , C. Cheng , Z. Liu , X. H. Zhang , Cell 2021, 184, 2471. 33878291 10.1016/j.cell.2021.03.011 PMC8087656

[30] T. Y. Ci , H. J. Li , G. J. Chen , Z. J. Wang , J. Q. Wang , P. Abdou , Y. M. Tu , G. Dotti , Z. Gu , Sci. Adv. 2020, 6, eabc3013. 33298439 10.1126/sciadv.abc3013 PMC7725453

