# Supplementary material for: Genetically Engineered Hematopoietic Stem Cells Deliver TGF‐β Inhibitor to Enhance Bone Metastases Immunotherapy
Source: Adv Sci (Weinh). 2022 Aug 10;9(28):2201451. doi: 10.1002/advs.202201451 (PMC9534984; doi:10.1002/advs.202201451)
Supplement: Supplementary file 1 — Supporting Information [file ADVS-9-2201451-s001.pdf]

ADVANCED SCIENCE
Open Access

Supporting Information

for *Adv. Sci.*, DOI 10.1002/advs.202201451

Genetically Engineered Hematopoietic Stem Cells Deliver TGF-β Inhibitor to Enhance Bone Metastases Immunotherapy

Beilei Wang, Jinyu Bai, Bo Tian, Hao Chen, Qianyu Yang, Yitong Chen, Jialu Xu, Yue Zhang, Huaxing Dai, Qingle Ma, Ziying Fei, Heng Wang, Fang Xu, Xiaozhong Zhou* and Chao Wang**

Supporting Information

Genetically Engineered Hematopoietic Stem Cells Deliver TGF-β Inhibitor to Enhance Bone Metastases Immunotherapy

Beilei Wang[1], Jinyu Bai[2]*, Bo Tian[2], Hao Chen[2], Qianyu Yang[1], Yitong Chen[1], Jialu Xu[1], Yue Zhang[1], Huaxing Dai[1], Qingle Ma[1], Ziying Fei[1], Heng Wang[1], Fang Xu[1], Xiaozhong Zhou[2]*, Chao Wang[1]*

[1] Institute of Functional Nano & Soft Materials, Soochow University, Suzhou, Jiangsu 215123, China.

[2] Department of Orthopedics, The Second Affiliated Hospital of Soochow University, Suzhou, Jiangsu 215004, China.

* Corresponding author: Chao Wang: cwang@suda.edu.cn, Xiaozhong Zhou: zhouxz@suda.edu.cn, Jinyu Bai: baijy@suda.edu.cn

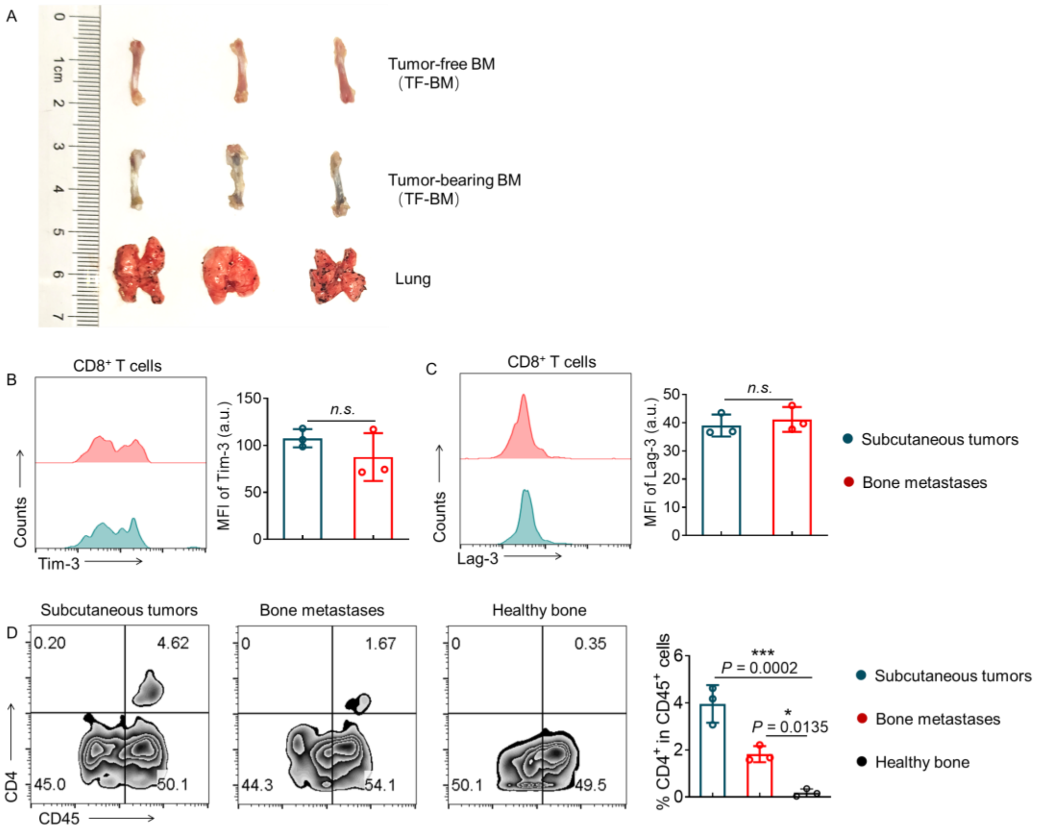

Figure S1. Construction of bone metastasis model and exploration of the immune microenvironment of bone metastasis. **A,** Construction of bone metastases models. Optical images of contralateral (right leg) tumor-free BM, tumor-bearing BM (left leg), and lung metastases. Photo credit: Beilei Wang, Soochow University. **B-C,** Comparison of the expression level of Tim-3 (B) and Lag-3 (C) in $CD8^+$ T cells in subcutaneous tumors and bone metastases. **D,** The proportions of $CD4^+$ T cells in subcutaneous tumors, bone metastases, and healthy bone. Data have been represented as mean ± SD. Statistical significance was calculated using Student's *t*-test and one-way ANOVA followed by Tukey's *post-hoc* test (n=3), *P*-value: $*P<0.05$, and $***P<0.001$; *n.s.*, no significance; a.u., arbitrary units; MFI, mean fluorescence intensity.

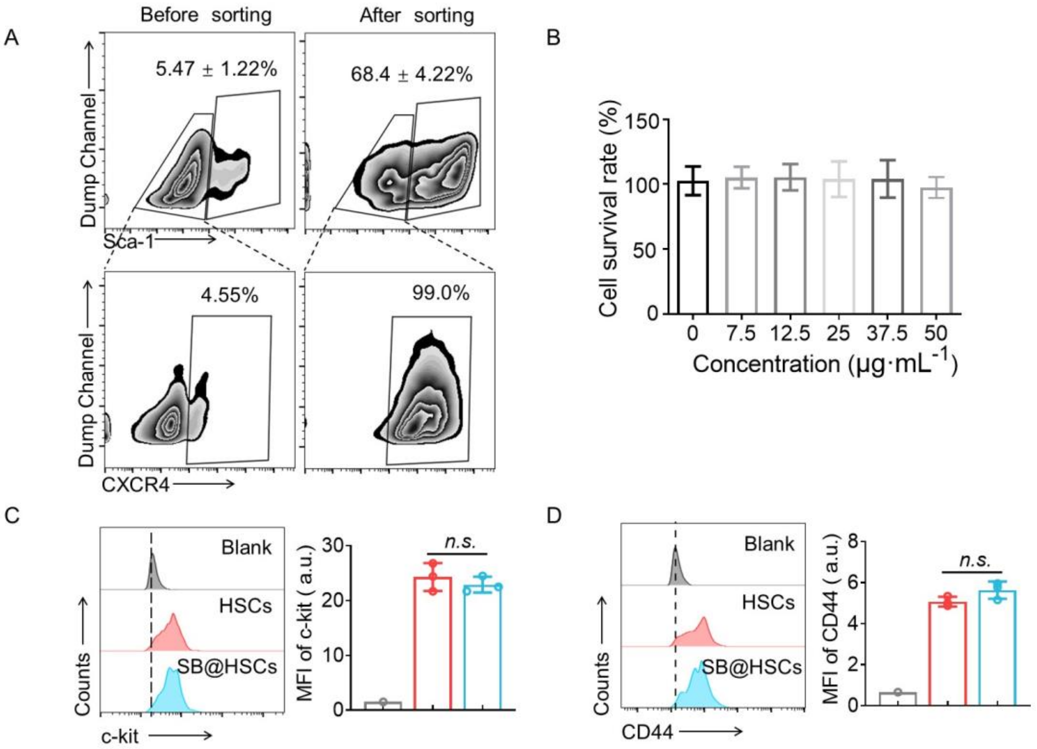

Figure S2. Characterization of HSCs loaded with TGF-β inhibitors. **A,** The purity of the HSCs was determined using flow cytometry. **B,** Cytotoxicity of SB, at different concentrations, in HSCs over 24 h (n=3). **C-D,** Expression levels of c-kit (C) and CD44 (D) in HSCs and SB@HSCs were analyzed using flow cytometry (n=3). Data have been represented as mean ± SD. Statistical significance was calculated using Student's t-test. *n.s.*, no significance; a.u., arbitrary units; MFI, mean fluorescence intensity.

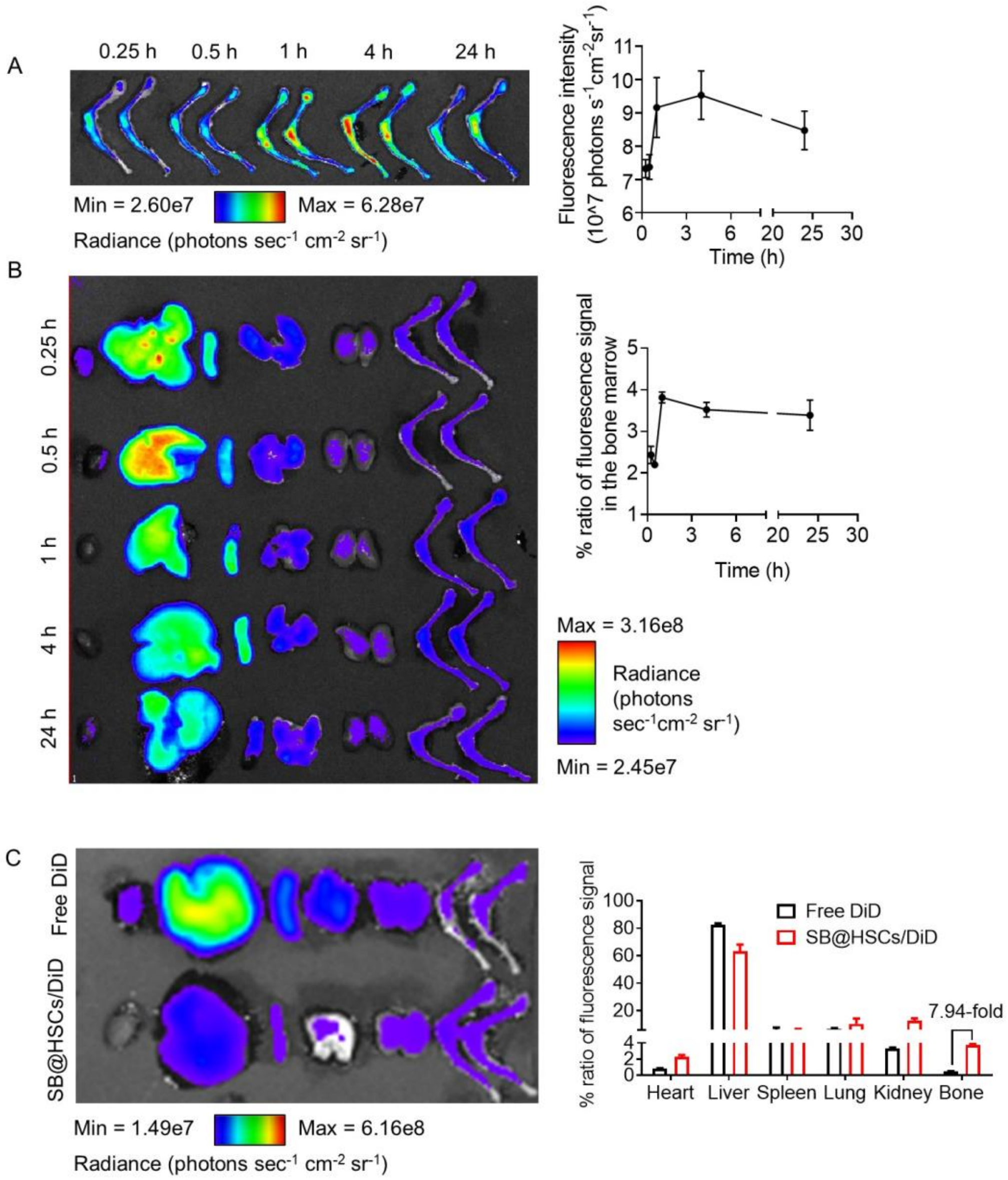

Figure S3. The homing of SB@HSCs in the bone marrow. **A,** Bone targeting of SB@HSCs at different time points. **B,** Homing efficiency of SB@HSCs at different time points. **C,** Homing efficiency of SB@HSCs after 24 h. Data have been represented as mean ± SD (n=3).

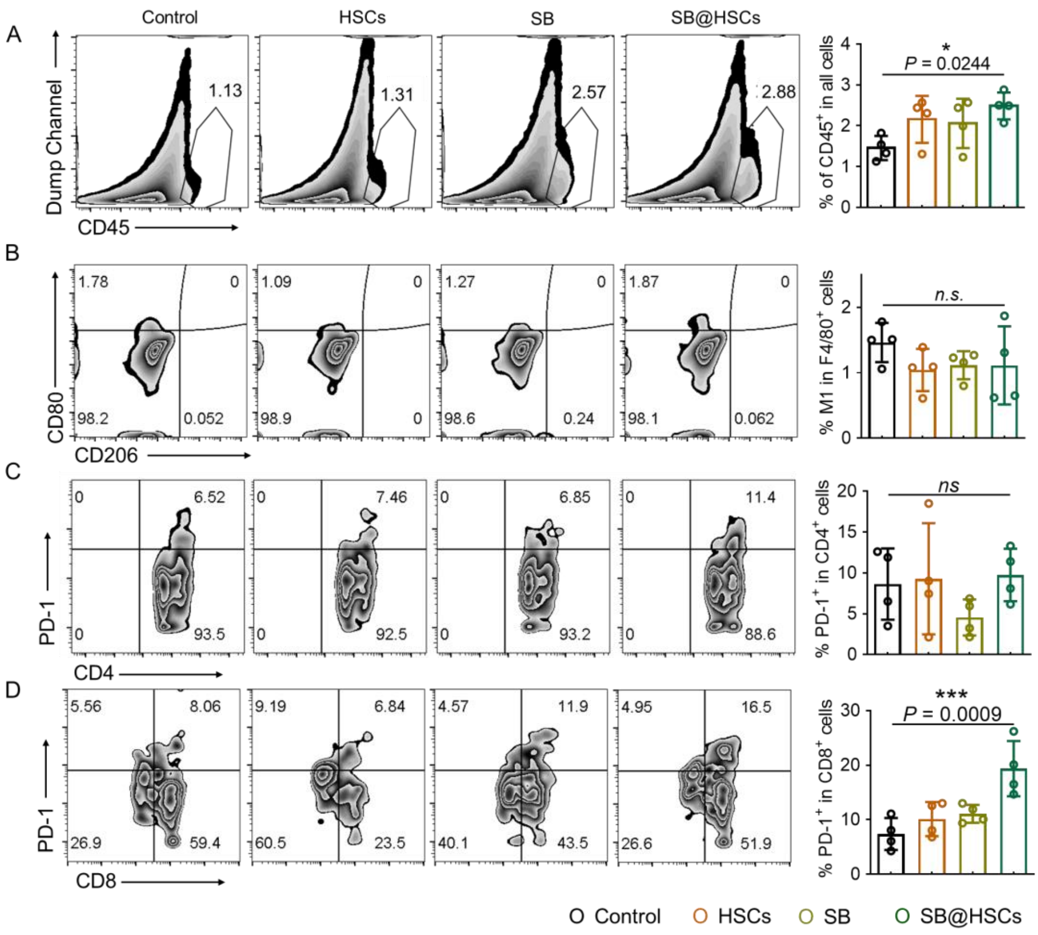

Figure S4. SB@HSCs reverse the immunosuppressive microenvironment in bone metastases. **A,** Representative flow cytometry zebra plots and statistical analyses of $CD45^+$ cells in mice subjected to different treatments. **B,** Representative flow cytometry zebra plots and statistical analyses of macrophages in mice subjected to different treatments. **C-D,** PD-1 expression in $CD4^+$ (C) and $CD8^+$ (D) T cells of different groups were analyzed using flow cytometry. Data have been represented as mean ± SD (n=4). Statistical significance was calculated using one-way ANOVA followed by Tukey's *post-hoc* test, *P*-value: **P*<0.05 and ****P*<0.001; *n.s.*, no significance.

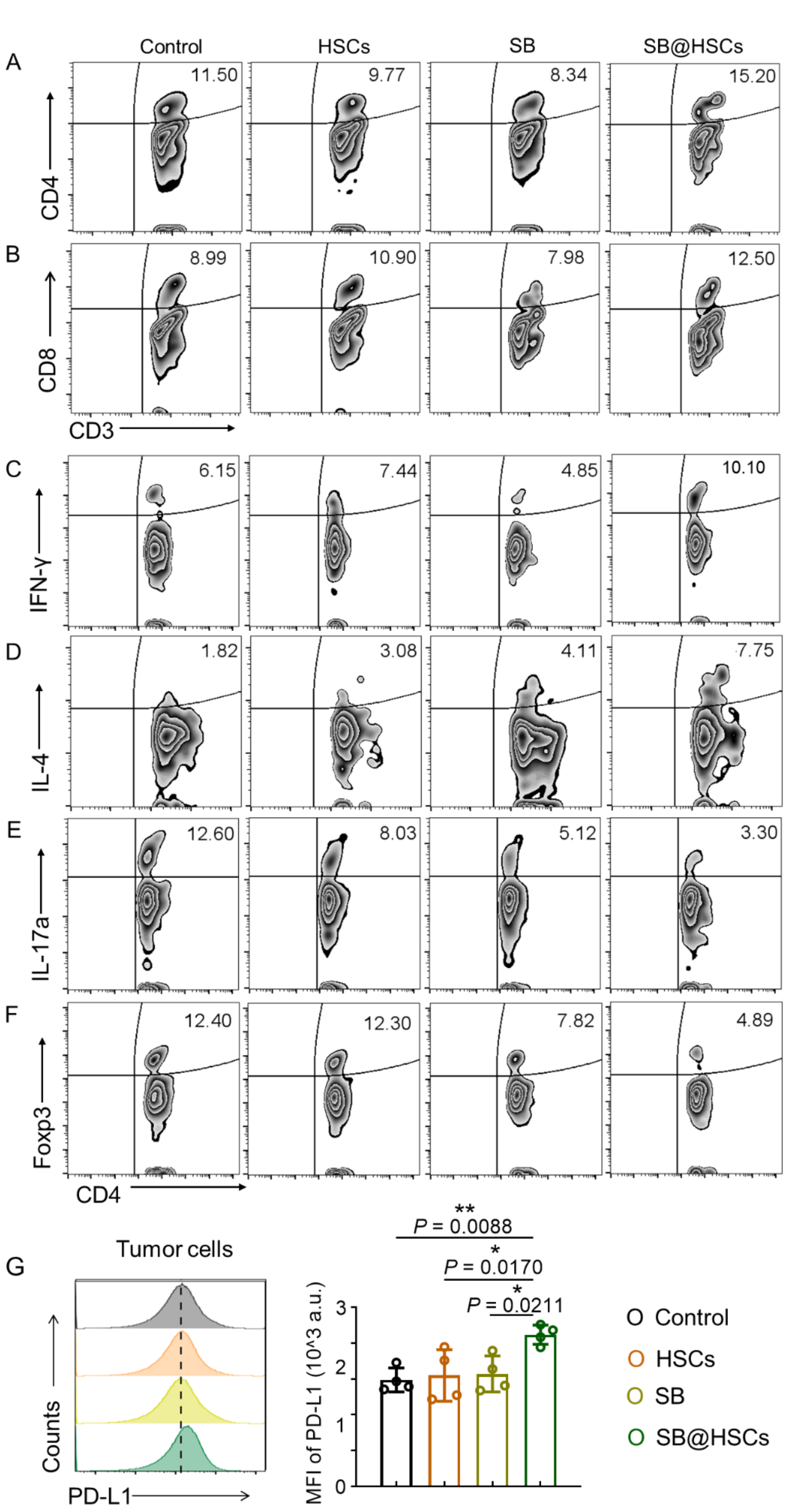

Figure S5. The immune microenvironment of bone metastases, after different treatments. **A–F,** Representative flow cytometry zebra plots of immune cells in mice:

$CD4^+$ T cells (A), $CD8^+$ T cells (B), T_H1 (C), T_H2 (D), T_H17 (E), and T_{reg} (F) cells. **G,** PD-L1 expression in tumor cells of all groups was analyzed using flow cytometry. Data have been represented as mean ± SD. Statistical significance was calculated using one-way analysis of ANOVA followed by Tukey's *post-hoc* test, *P*-value: **P*<0.05 and ***P*<0.01; a.u., arbitrary units; MFI, mean fluorescence intensity.

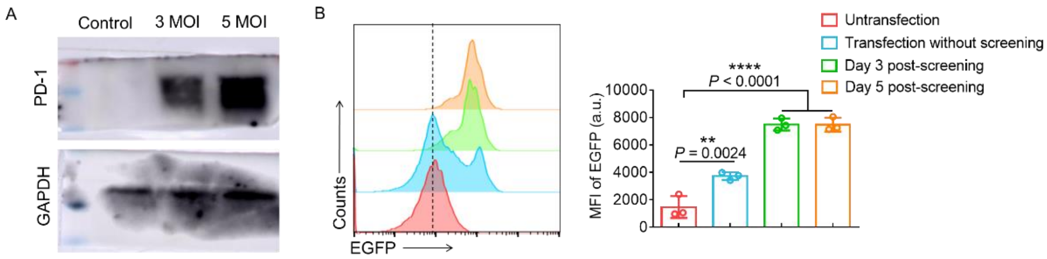

Figure S6. Lentiviral transfection of HSCs. **A,** Original uncropped images with PD-1 (52 kDa) and GAPDH (37 kDa) markers. **B,** We used 5 μg/mL puromycin to screen stable transgenic strains, and EGFP signal of HSCs-PD-1 was measured using flow cytometry (n=3). Data have been represented as mean ± SD. Statistical significance was calculated using one-way analysis of variance followed by Tukey's *post-hoc* test, *P*-value: ***P*<0.01 and *****P*<0.0001. a.u., arbitrary units; MFI, mean fluorescence intensity.

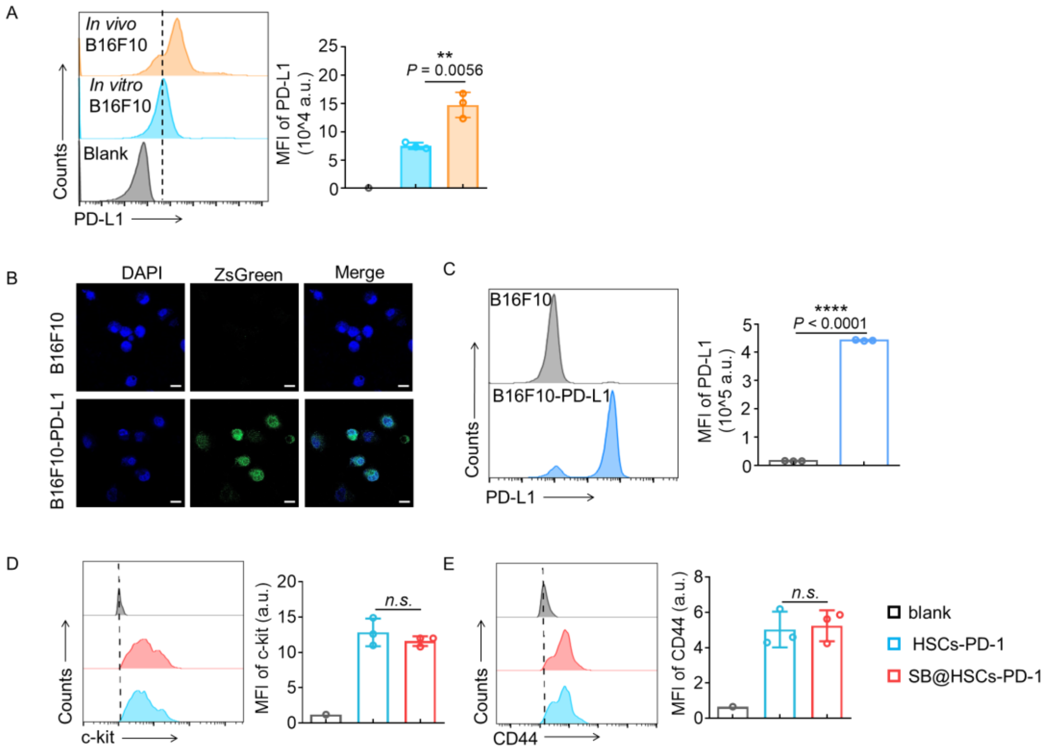

Figure S7. Lentivirus transfection of B16F10 cells. **A,** Comparison of PD-L1 expression levels between B16F10 cells in bone metastasis and B16F10 cells cultured *in vitro*. **B,** Confocal images of B16F10 and B16F10-PD-L1 cells. Cell nucleus were stained with DAPI (blue). Scale bar: 10 μm. **C,** Transduction efficiency was measured using flow cytometry (n=3). **D-E,** Expression levels of c-kit (D) and CD44 (E) in HSCs-PD-1 and SB@HSCs-PD-1 were analyzed using flow cytometry (n=3). Data have been represented as mean ± SD. Statistical significance was calculated using Student's t-test, *P*-value: ***P*<0.01 and *****P*<0.0001; a.u., arbitrary units; MFI, mean fluorescence intensity.

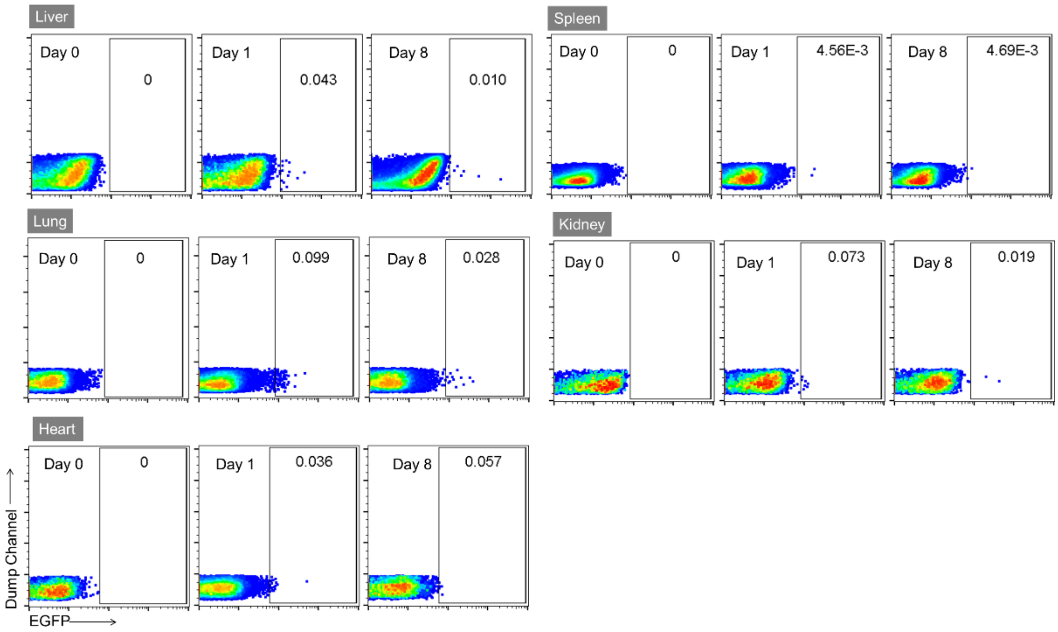

Figure S8. *In vivo* biodistribution of HSCs-PD-1. Representative flow cytometric images of $EGFP^+$ cells in major organs, on 0th, 1st, and 8th days after treatment with $EGFP^+$ HSCs.

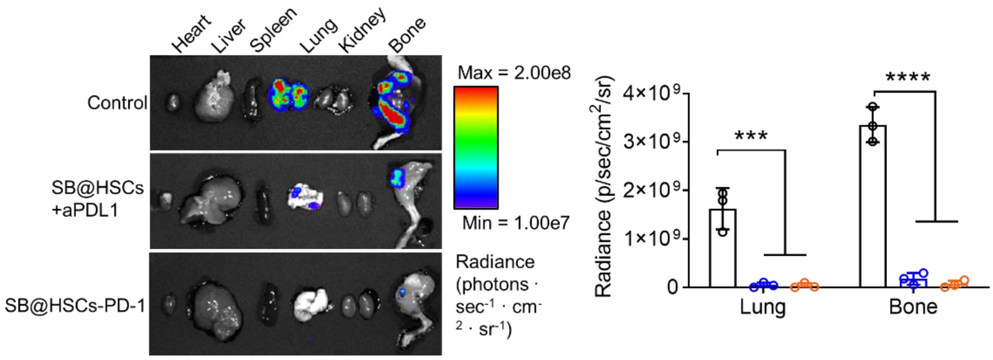

Figure S9. IVIS images® and fluorescence intensities of typical organs on the 18th day after bone metastasis seeding. Statistical significance was calculated using one-way ANOVA followed by Tukey's *post-hoc* test (n=3), *P*-value: ***P<0.001 and ****P<0.0001.

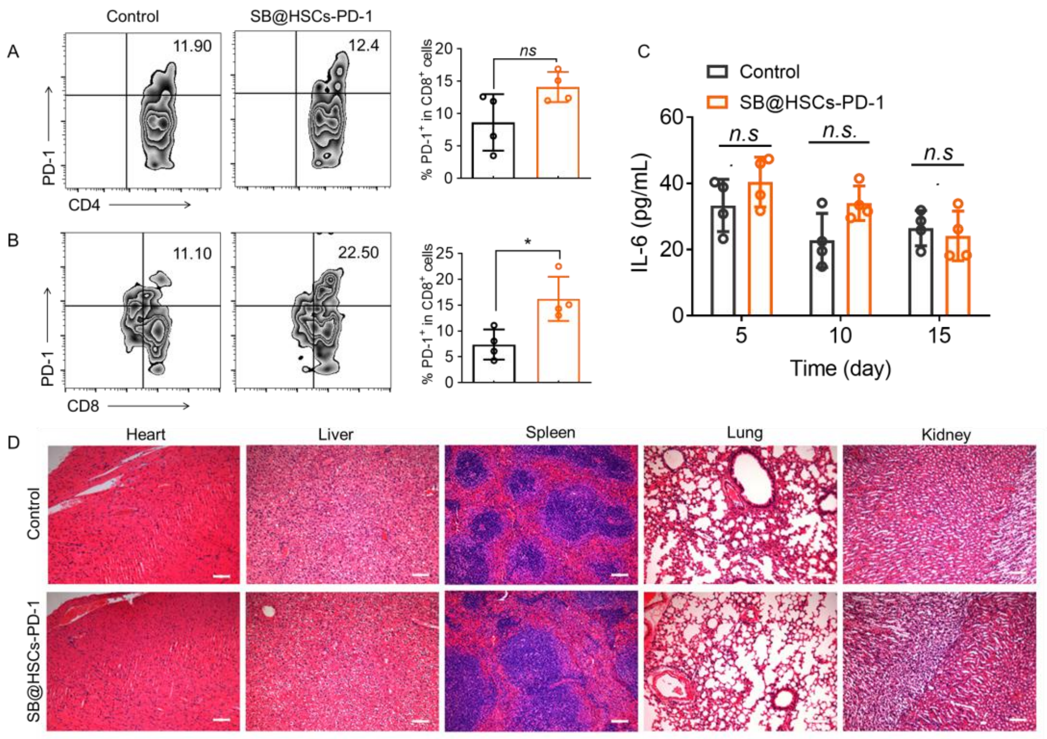

Figure S10. The immune microenvironment of bone metastases after HSCs-PD-1@SB treatments and the safety of HSC-PD-1@SB. **A-B,** PD-1 expression in $CD4^+$ (A) and $CD8^+$ (B) T cells after HSCs-PD-1@SB treatments was analyzed using flow cytometry (n=4). **C,** IL-6 levels in the serum of mice on day 5, 10, and 15, after the different treatments (n=4). **D,** Heart, liver, spleen, lung, and kidney of mice without or with SB@HSCs-PD-1 treatment were sectioned and stained (H&E) for histological analysis. Scale bar: 100 μm. Data have been represented as mean ± SD. Statistical significance was calculated using the Student's *t*-test, *P*-value: *$P<0.05$; *n.s.*, no significance.
